# Breath aerosol PCR for detection of lower respiratory tract infections: evaluation of a non-invasive face mask collector in pneumonia patients

**DOI:** 10.1128/jcm.00562-26

**Published:** 2026-09-10

**Authors:** Katie Tiseo, Sarah Dräger, Harshitha Santhosh Kumar, Maia Alkhazashvili, Anya Hammann, Patricia Risch, Reto Willi, Tamar Mkhatvari, Christina Fialová, Christian Adlhart, Denise Szabó, Mariam Suknidze, Izolda Pachkoria, Tobias Broger, Elena Ivanova Reipold, Khatuna Varshanidze, Michael Osthoff

**Affiliations:** 1 Avelo AG, Schlieren, Switzerland; 2 Department of Internal Medicine, University Hospital Basel 30262 , Basel, Switzerland; 3 National Center for Disease Control and Public Health of Georgia 227253, Tbilisi, Georgia; 4 Avicenna – Batumi Medical University, Batumi University Hospital (MedCenter), Batumi, Georgia; 5 ZHAW Zurich University of Applied Sciences, School of Life Sciences and Facility Management, ZHAW University of Applied Sciences Institute of Chemistry and Biotechnology 537292 , Wädenswil, Switzerland; 6 Avicenna – Batumi Medical University, Batumi, Georgia; 7 Hightechnology Hospital Medcenter, Batumi, Georgia; 8 Department of Internal Medicine, Cantonal Hospital Winterthur 30934 , Winterthur, Switzerland; 9 Department of Clinical Research, University of Basel 27209, Basel, Switzerland; National Institute of Allergy and Infectious Diseases Division of Intramural Research, Bethesda, Maryland, USA

**Keywords:** lower respiratory tract infection, PCR, breath, aerosol, non-sputum, diagnosis, pneumonia

## Abstract

Etiological diagnosis of lower respiratory tract infections (LRTIs) relies on sputum or bronchoalveolar lavage (BAL), which may be difficult to obtain or invasive. Exhaled breath aerosol (XBA) sampling offers a noninvasive alternative for pathogen detection. We evaluated the performance of the AveloMask, a face mask-based device designed to capture XBAs for molecular testing. In this prospective paired-sample study, hospitalized adults with pneumonia at three hospitals in Switzerland and Georgia provided an XBA sample using the AveloMask and a lower respiratory tract (LRT) specimen (sputum or BAL). XBA samples were analyzed by multiplex PCR using the Roche LightMix® panel, and LRT samples were tested using the BioFire® FilmArray® Pneumonia Panel. Concordance between XBA and LRT samples was assessed using positive percent agreement (PPA), negative percent agreement (NPA), and overall percent agreement (OPA). A total of 93 participants were enrolled, and 63 participants provided paired samples. AveloMask sampling identified the dominant pathogen (lowest Ct value in the LRT sample) in 40/47 LRT-positive cases (85.1%). Across all targets, PPA was 61% (95% CI, 50%–72%), NPA was 99.8% (95% CI, 99%–100%), and OPA was 95% (95% CI, 92%–96%). PPA was higher for bacteria than for viruses, and lower PPA was largely driven by reduced detection of low-abundance or co-infecting pathogens. In an exploratory subset analysis, AveloMask results overlapped with standard-of-care testing and may have informed antimicrobial decision-making. Breath aerosol sampling using the AveloMask enabled noninvasive molecular detection of LRT pathogens in pneumonia cases and may complement conventional standard-of-care testing, particularly when sputum is unavailable.

**IMPORTANCE:**

Pneumonia diagnosis often relies on sputum samples, which many patients cannot provide, or bronchoscopy, which is invasive. This study evaluated a user-friendly face mask that captures exhaled aerosols released during normal breathing and coughing for laboratory testing. In hospitalized patients with pneumonia, the mask identified the most abundant pathogen in most cases and was well tolerated in routine clinical care. These findings show that breath aerosol sampling could provide a practical, noninvasive complement to existing diagnostic methods, especially when sputum is unavailable or difficult to obtain. By making lower respiratory tract (LRT) sampling easier and more accessible, this approach could support faster, more targeted treatment decisions, reduce unnecessary antibiotic use, and expand opportunities for molecular diagnosis in hospitals and other healthcare settings.

This study is registered with ClinicalTrials.gov as NCT06668883.

## INTRODUCTION

Lower respiratory tract infections (LRTIs) causing pneumonia are a leading cause of morbidity and mortality worldwide and account for a substantial proportion of hospital admissions and antibiotic use (1–3). Accurate identification of the causative pathogen is essential to guide targeted therapy, inform infection control measures, and reduce unnecessary antibiotic use. Multiplex PCR assays have improved the sensitivity and speed of pathogen detection from respiratory specimens compared with conventional culture-based methods; however, the clinical utility of multiplex PCR depends heavily on specimen quality and type. Conventional diagnostic samples for LRTIs include sputum and bronchoalveolar lavage (BAL). High-quality sputum specimens can be difficult to obtain, and BAL is invasive and resource-intensive, limiting its routine use (4, 5). While upper respiratory tract swabs are easier and faster to collect, they may not reliably reflect pathogens causing disease in the lower respiratory tract (LRT) (6, 7). These limitations can result in diagnostic uncertainty, encourage empiric broad-spectrum antibiotic prescribing, and may impair timely de-escalation. The ongoing debate in pneumonia guidelines—including recommendations for antibiotic use in patients with confirmed viral infections—highlights the need for improved, noninvasive diagnostic approaches that enable timely pathogen detection and more individualized antimicrobial decisions (8).

Noninvasive sampling of exhaled breath aerosols (XBAs) represents a potential complement to traditional respiratory specimens. Respiratory pathogens are known to be transmitted via aerosols, and studies have demonstrated that viruses and bacteria can be detected in XBAs (9–12). However, much of the prior work on XBAs has either focused on surrogate biomarkers such as volatile organic compounds (VOCs), which often have low specificity, or has used large equipment in research settings that is impractical for routine clinical workflows (13–16).

The AveloMask (Avelo AG, Switzerland) is a face mask-based breath sampling kit designed to capture XBAs during normal breathing and coughing onto an integrated filter. They are subsequently transferred into a stabilization buffer for downstream nucleic acid detection. This approach offers a scalable, noninvasive complement to traditional LRT sampling, particularly when sputum is unavailable or of poor quality. In this prospective, paired-sample study in hospitalized adults with radiographically confirmed pneumonia, we evaluated the diagnostic performance of the AveloMask by comparing pathogen detection in XBA samples with that in standard LRT specimens (sputum or BAL) using commercial multiplex PCR.

## MATERIALS AND METHODS

### AveloMask Kit design and procedure

**Fig 1:**
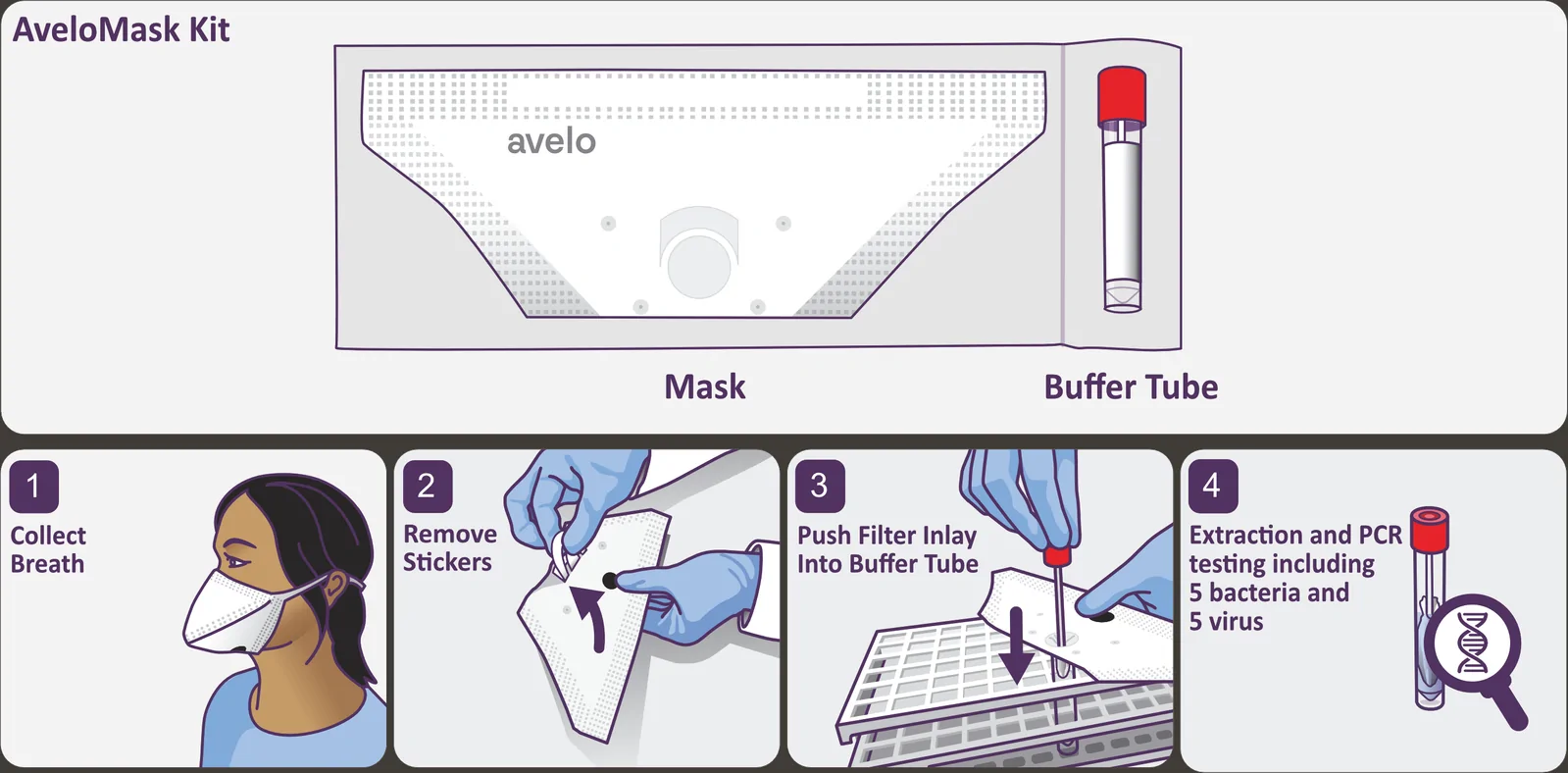
AveloMask Kit and breath aerosol collection and testing procedure. Figure adapted from Risch et al. (10).

The AveloMask (Avelo AG, Switzerland) is a specimen collection kit designed to collect XBA samples from the respiratory tract. The processing steps are illustrated in Fig. 1. The kit consists of a face mask and a buffer tube. The inner surface of the mask contains an electrospun fiber filter inlay that captures the aerosolized particles from exhaled breath (10).

After wearing the mask (step 1), two protective stickers on either side of the mask are removed (step 2). The filter inlay is then transferred into the buffer tube by pushing it into the tube using a stick attached to the tube cap (step 3). The buffer inactivates the sample and preserves nucleic acids, allowing transport at ambient temperature until further processing or biobanking (step 4). Detailed instructions are provided in Movie S1 and Fig. S1 (10).

### Clinical evaluation

This prospective, paired-sample comparison study initially enrolled participants from an inpatient hospital site in Basel, Switzerland (clinicaltrials.gov, NCT06668883). It was subsequently expanded to include two hospital sites in Batumi, Georgia, to achieve the target sample size, using closely aligned study procedures across sites. Participants were enrolled between 13 November 2024 and 12 June 2025. Eligible participants were consenting adults (≥18 years of age) with a diagnosis of LRTI defined as a new pulmonary infiltrate on chest imaging and at least one clinical symptom consistent with an LRTI, including fever, chills, cough, sputum production, dyspnea, tachypnea, or chest pain (Basel only). Participants were excluded if they were unable to complete all sampling procedures within 48 h of initiating new antibiotic therapy; were critically ill at the discretion of the investigator; or were receiving oxygen supplementation via a face mask, high-flow oxygen, noninvasive ventilation, or mechanical ventilation. Participants receiving supplemental oxygen via a nasal cannula were eligible for inclusion.

### Sampling methods

Demographic and clinical data were collected using case report forms (CRFs). In Basel, data were recorded electronically using secuTrial, a GCP-compliant electronic data capture system (interActive Systems GmbH, Berlin [Germany]), whereas paper CRFs were used in Batumi.

Sputum collection was attempted for all participants and was performed either spontaneously or via induction according to standard clinical procedures. Sputum samples were required to appear visually mucoid or purulent at the time of collection, and samples meeting this criterion underwent further laboratory assessment. Those containing <25 squamous epithelial cells per low-power field were considered of acceptable quality. Acceptable sputum samples were used as the comparator LRT specimen and were stored at −80°C until shipment to Avelo AG (Switzerland) for batch testing. BAL samples were not collected as a part of this study; however, when BAL was collected as a part of routine clinical care and when no acceptable sputum sample was available, BAL samples were permitted as the comparator LRT specimen.

For breath sampling, participants wore the AveloMask for 30 min and were instructed to perform 20 deep coughs in addition to any spontaneous coughs during mask wear. Mask samples were processed onsite by trained study nurses. Total hands-on time for the study nurse was approximately 3 min. Filter inlays were transported in stabilizing buffer at ambient temperature on the day of collection, stored at −80°C, and shipped on dry ice to Avelo AG for batch testing. All samples were stored at −80°C for up to 6 months prior to testing.

To gather basic feedback on ease of use, participants were asked immediately after sample collection: “Overall, how difficult or easy was it to provide the AveloMask sample?” Answers were recorded on a 5-point scale and recorded in the CRFs.

### PCR methods

#### AveloMask samples

Frozen AveloMask samples, consisting of filter inlays submerged in stabilizing buffer, were thawed and briefly centrifuged for 10 s at 2,000 rcf prior to opening to prevent aerosol contamination. The recommended amount of DNA and Equine Arteritis Virus (EAV) RNA process controls (Roche, Switzerland) and 30 µg of carrier RNA were added to each tube, followed by vortexing for 10 s and incubation in a water bath at 70°C for 15 min. Following heat elution and lysis, the tube was vortexed for additional 10 s and centrifuged at 2,000 rcf for 10 s.

Then, the filter inlay was compressed using a disposable sterile Pasteur pipette, and the eluate was transferred to a fresh tube for nucleic acid extraction. Molecular-grade ethanol (2.0 mL) was added, and DNA and RNA were extracted from the entire sample using the QIAamp DNA Mini Kit (Qiagen, Germany) according to the manufacturer’s instructions, with omission of proteinase K and Buffer AL, as the sample buffer already contained guanidinium thiocyanate. Nucleic acids were eluted in 100 μL of Buffer AE.

Each extraction batch included a negative control consisting of buffer containing DNA and EAV RNA process controls and 30 µg carrier RNA, which was required to be negative for all pathogen targets while demonstrating appropriate amplification of process controls for run validity. For each sample, two 10-μL aliquots of the extract were analyzed, one for viral targets and one for bacterial targets. Quantitative PCR was performed on a LightCycler® 480 II instrument (Roche, Switzerland) using modular Roche LightMix® primer and probe sets targeting five viruses (influenza A virus, influenza B virus, respiratory syncytial virus (RSV), SARS-CoV-2, and rhinovirus), along with the EAV RNA process control, and five bacteria (*Mycoplasma pneumoniae, Chlamydia pneumoniae, Haemophilus influenzae, Streptococcus pneumoniae, and Legionella pneumophila*), together with the DNA process control. Separate RNA and DNA master mixes were used as required.

LightMix modular positive controls for all pathogens were included in each reaction plate. Any amplification curve consistent with a sigmoidal (S-shaped) amplification curve crossing the fluorescence threshold within the specified Ct range for the target and a Ct value <45 was considered positive. In cases where no pathogens were detected, process controls were checked to confirm assay validity. Samples were called invalid if both pathogens and process controls were negative. Samples were called indeterminates when their amplification curves did not exhibit a typical sigmoidal morphology. Invalid runs and indeterminate samples were repeated where sufficient sample material was available. Samples remaining invalid or indeterminate after repeat testing were excluded from the final analysis. Laboratory personnel performing mask sample testing were blinded to participant clinical information.

#### LRT samples

Comparator LRT samples were tested using the Biofire® FilmArray® Pneumonia Plus Panel (BioMérieux, France), a CE-IVD-marked point-of-care multiplex PCR assay validated for sputum and BAL samples. This platform was selected as the reference method because it is widely implemented in routine clinical practice, is validated for LRT specimens, and provides standardized, automated results with minimal operator-dependent variability. Testing was performed according to the manufacturer’s instructions, and results were interpreted based on the automated report generated at completion of each run. In addition, 200 µL of the residual LRT sample was extracted and tested using the LightMix panel following a similar protocol as for AveloMask samples. The LightMix and BioFire panels overlapped for all targets included in the LightMix panel, except for SARS-CoV-2. Laboratory personnel performing LRT sample testing were blinded to participant clinical information but were not blinded to the LRT comparator results. This reflected the centralized testing workflow and limited laboratory staffing.

### Data analysis

Sample size was determined using a precision-based approach, targeting two-sided Wilson score confidence intervals with a width of ±15%, assuming a positive percent agreement (PPA) of 70% for mask-based qPCR relative to LRT samples across pathogens. PPA was used for sample size determination as fewer positive pathogen agreement events are expected compared to negative agreement events in the PCR panel. Assuming detectable pathogens in at least 50% of LRT samples based on previous studies (17) and an indeterminate test rate of 8% yielded a sample size of 60 participants with 55 evaluable paired XBA and LRT samples.

Point estimates and 95% Wilson score confidence intervals were calculated for PPA, negative percent agreement (NPA), and overall percent agreement (OPA) of AveloMask LightMix PCR panel results compared with LRT BioFire PCR panel results. Agreement analyses were conducted per participant, per pathogen, and across all pathogens combined and stratified by viral and bacterial targets, study site, and LRT BioFire sample Ct value. The same analyses were repeated for AveloMask LightMix PCR versus LRT LightMix PCR in a secondary analysis. We reported pathogen positivity of AveloMask versus LRT samples using heatmaps and performed an analysis to determine the percentage of participants in whom AveloMask detected the dominant LRT pathogen (defined as the pathogen with the lowest Ct value in the LRT specimen). Missing and invalid results were reported, and agreement analyses were restricted to complete paired samples with valid index and reference test results. In a subset of participants enrolled in Basel, AveloMask LightMix PCR, LRT BioFire, and available standard-of-care microbiological testing results were descriptively compared to assess pathogen detection overlap using Euler diagrams. In addition, the principal investigator and an independent clinically trained infectious disease consultant performed an exploratory *post hoc* assessment of the potential impact of AveloMask results on antimicrobial management based on clinical information and standard-of-care test (SOCT) results available at the time of care. Assessments were performed in the context of local antimicrobial stewardship practices and were considered exploratory rather than formal clinical adjudications. All analyses were performed using R (version 4.5.0) (18).

## RESULTS

### Participant characteristics

**Fig 2:**
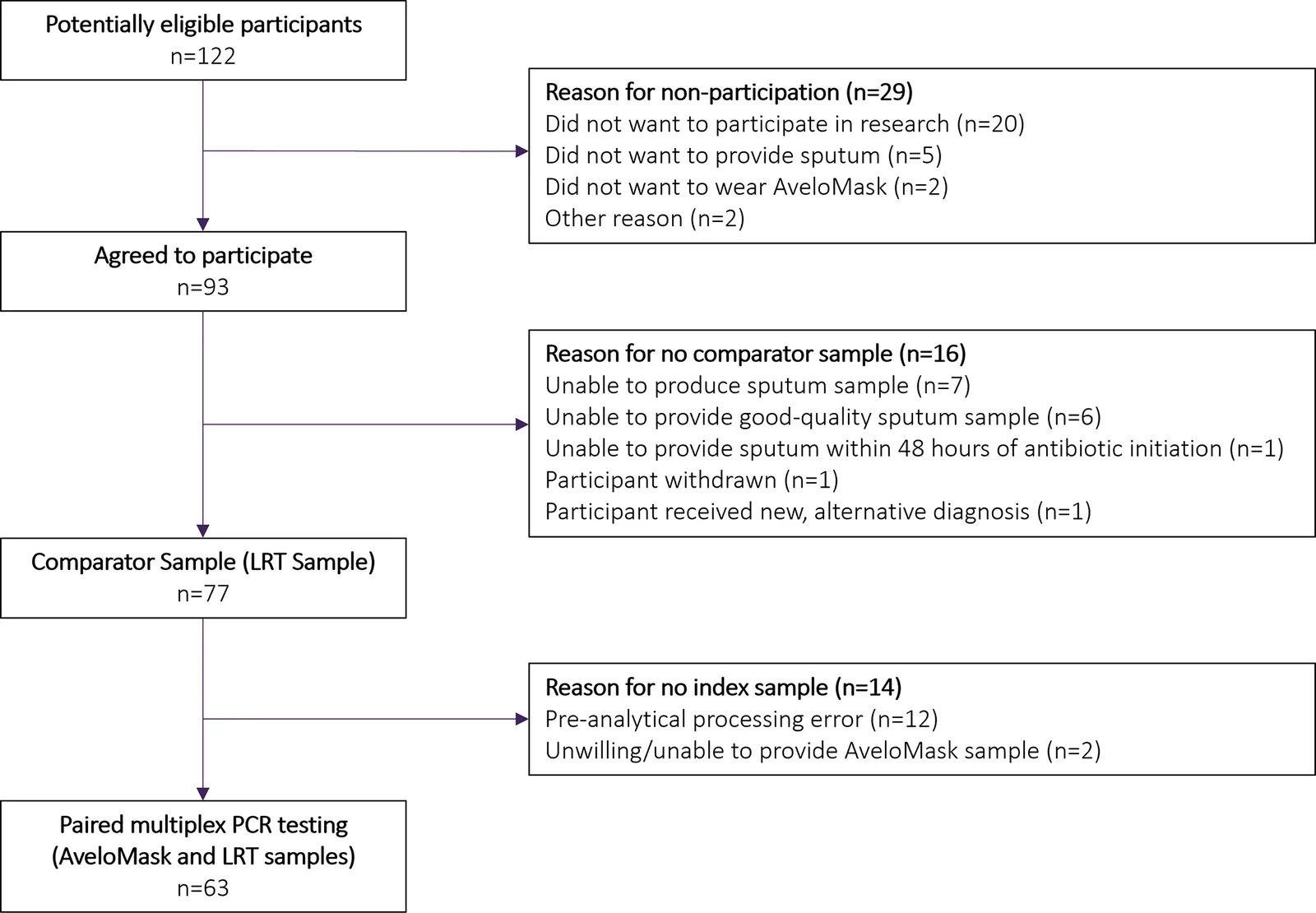
Study flowchart. Abbreviation: LRT, lower respiratory tract.

Of the 122 eligible participants, 93 consented to participate (Fig. 2). Thirteen participants (14.3%) were excluded due to issues with sputum production (seven were unable to produce any sputum, and six had samples of insufficient quality). Fourteen AveloMask samples were excluded prior to PCR analysis due to pre-analytical processing errors (*n* = 12) and participants being unable to provide AveloMask samples (*n* = 2) (Table S1). Ultimately, 63 participants who successfully provided an LRT sample and an AveloMask XBA sample were included. Comparator LRT specimens consisted of spontaneously produced sputum (58/63, 92.1%), induced sputum (4/63, 6.3%), and one BAL sample (1/63, 1.6%). Most participants (57/63, 90.5%) had initiated antibiotic therapy prior to sample collection. Demographic and clinical characteristics are summarized in Table 1. The median time from antibiotic initiation to AveloMask sampling was 22 h (range, −1 to 44 h). The median time interval between collection of the two sample types was 1 h (range, 2 min to 25 h). The study population primarily consisted of middle-aged adults, with a slight predominance of males. No adverse events or complications were reported.

**TABLE 1:** Characteristics of the study cohort^*a*^

Characteristics	*n* = 63
Age (years)	63 (19–88)
Sex, female	23 (37)
Immunocompromising condition or treatment^*b*^	11 (17)
Clinical and laboratory findings on admission	
Fever	34 (54)
Chills	5 (8)
Cough	54 (86)
New or worsening sputum production	25 (40)
Dyspnea	14 (22)
Tachypnoea	9 (14)
CRP (mg/L) (*n* = 62/63)	68 (1–406)
White blood cell count (g/L)	10.5 (2.3–31.1)
Respiratory rate (*n* = 60/63)	30 (14–40)
Oxygen saturation (% SpO_2_)	90 (80–99)
Heart rate	124 (60–151)
Sample timing	
Hours between antibiotics (if any) and AveloMask sampling (*n* = 59/63)	22 (−1 to 44)^*c*^
Severity index scores	
CURB-65 score	2 (0–4)
Charlson comorbidity index	3 (0–9)

^*a*^ Continuous data are presented as median (range) and categorical data as counts (relative percentages). CRP, C-reactive protein; CURB-65, confusion, urea, respiratory rate, blood pressure, and age ≥ 65 years.

^*b*^ Immunocompromising condition or treatment was defined as a hematological disease, an oncological disease, human immunodeficiency virus (HIV), hemodialysis, solid organ transplantation, stem cell transplantation, currently undergoing chemotherapy, on chronic oral steroids, and/or on immunosuppressant drugs.

^*c*^ Range begins at −1 as one patient started antibiotic treatment post-AveloMask collection.

### Per participant concordance

**Fig 3:**
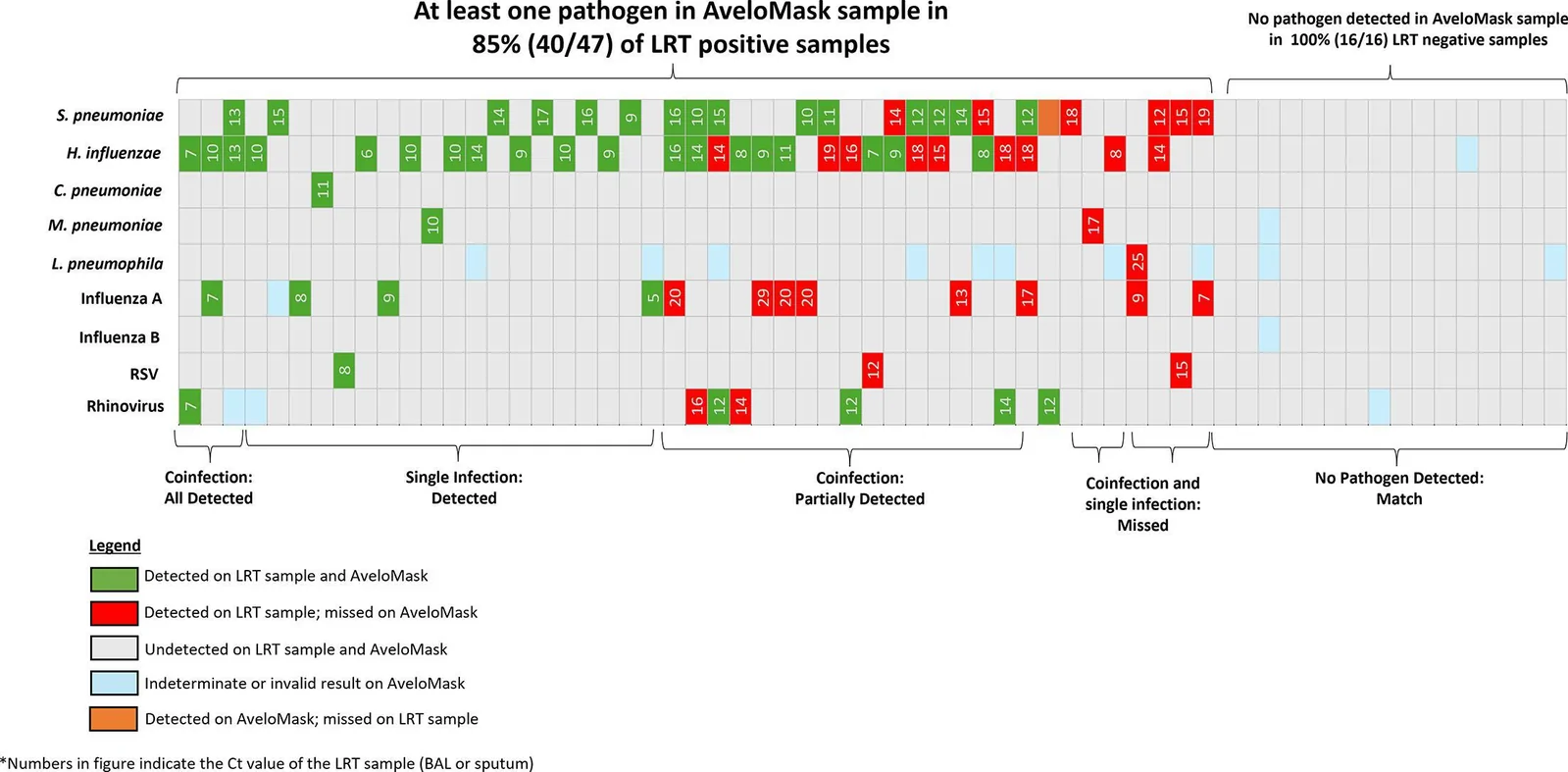
Heat map for per participant concordance between paired LRT BioFire and AveloMask LightMix PCR results. The numbers indicated in the figure show the Ct values of the LRT sample using the BioFire panel. Abbreviations: LRT, lower respiratory tract; RSV, respiratory syncytial virus.

Overall, 47 participants (74.6%) had at least one overlapping-panel pathogen identified (Fig. 3). Considering only pathogens included on both the LightMix and BioFire panels, 23 participants (36.5%) had a single pathogen detected in their LRT sample, 24 (38.1%) had coinfections, and 16 (25.4%) had no pathogen detected. Among participants with no overlapping pathogen detected in LRT samples (*n* = 16), alternative microbiological diagnoses were established for 10 participants using LRT BioFire results for non-overlapping pathogens. Pathogens included *Staphylococcus aureus* (*n* = 2), *Streptococcus agalactiae* (*n* = 2), human metapneumovirus (*n* = 2), adenovirus (*n* = 2), *Klebsiella pneumoniae* (*n* = 1), rhinovirus (in a case with an indeterminate AveloMask result [*n* = 1]), and human coronavirus (*n* = 1). Six participants had no etiology identified. Although SARS-CoV-2 was included in the LightMix panel, there were no detections in either sample type.

In participants with multiple pathogens detected in their LRT sample, the pathogen with the lowest Ct value in the LRT sample (and therefore highest abundance, subsequently called the “dominant pathogen”) was consistently the pathogen detected in the paired AveloMask sample. AveloMask sampling detected the dominant pathogen in 40/47 (85.1%, 95% CI, 72%–93%) LRT-positive participants (Fig. 3). Among seven participants with pathogen-positive LRT samples and no corresponding detection by AveloMask sampling, a total of 11 pathogens were identified in the LRT samples. Eight of 11 pathogens were detected at high Ct values (>12) in the BioFire panel, suggesting relatively low pathogen abundance. Ct values reported by BioFire are lower than conventional qPCR Ct values as the assay employs a nested, multiplex design. One additional missed pathogen had a low Ct value (*H. influenzae*, Ct 8) but occurred in a coinfection with human coronavirus, which was detected at a lower Ct value and was not part of the LightMix panel. The remaining missed pathogens were both influenza A detected in LRT samples at relatively low Ct values (7 and 9), with no evidence of other dominant pathogens in the corresponding LRT samples. The timing between antibiotic initiation and AveloMask sampling in these seven participants (median, 22 h; range, 0–35 h) was similar to that observed in the overall study population.

One participant tested positive for *S. pneumoniae* by AveloMask sampling but tested negative in their paired LRT sample. Review of SOCT data revealed a positive pneumococcal urine antigen test and a positive sputum culture in this participant, supporting the presence of *S. pneumoniae* infection.

Pathogens detected by AveloMask sampling were associated with lower LRT Ct values on the BioFire panel (mean Ct, 11), whereas pathogens undetected by AveloMask had higher Ct values in their corresponding LRT samples (mean Ct, 16).

### Per-pathogen and overall concordance

The overall concordance between AveloMask and LRT samples is summarized in Table 2. *H. influenzae* and *S. pneumoniae* were the most frequently detected bacterial pathogens, with PPAs of 68% and 71%, respectively. Rhinovirus was the most common viral pathogen with a PPA of 71%. Lower PPAs were observed for influenza A and RSV (33%). The number of RSV-positive LRT samples was limited (*n* = 3), whereas influenza A was frequently detected in LRT samples but less consistently identified in AveloMask samples. Across shared targets, AveloMask sampling demonstrated an overall PPA of 61% (95% CI, 50%–72%), NPA of 99.8% (95% CI, 99%–100%), and OPA of 95% (95% CI, 92%–96%) compared with LRT samples. Across 563 pathogen-level comparisons (9 targets in 63 participants), 17 (3.0%) AveloMask LightMix results were invalid (*n* = 10) or indeterminate (*n* = 7).

**TABLE 2:** Per-pathogen and overall concordance between detections for paired LRT BioFire samples and AveloMask LightMix samples (*n* = 63)^*a*^

Pathogen	AveloMask- and LRT-positive	Only LRT-positive	Only AveloMask-positive	Total negative	OPA	PPA	NPA	PPV	NPV
Bacteria									
*Streptococcus pneumoniae*	15	6	1	41	89%	71%	98%	94%	87%
*Haemophilus influenzae*	19	9	0	34	85%	68%	100%	100%	79%
*Chlamydia pneumoniae*	1	0	0	62	100%	100%	100%	100%	100%
*Mycoplasma pneumoniae*	1	1	0	60	98%	50%	100%	100%	98%
*Legionella pneumophila*	0	1	0	52	98%	0%	100%	NA*^b^*	98%
Virus									
Influenza A	4	8	0	50	87%	33%	100%	100%	86%
Influenza B	0	0	0	62	100%	NA	100%	NA	100%
Respiratory syncytial virus (RSV)	1	2	0	60	97%	33%	100%	100%	97%
Rhinovirus/Enterovirus	5	2	0	53	97%	71%	100%	0%	98%
Overall	46	29	1	474	95%	61%	99.8%	98%	94%
Lower 95% CI					92%	50%	99%		
Upper 95% CI					96%	72%	100%		

^*a*^ LRT, lower respiratory tract; OPA, overall percent agreement; PPA, positive percent agreement; NPA, negative percent agreement; PPV, positive predictive value; NPV, negative predictive value.

^*b*^ NA, not applicable; the denominator of the equation was 0 because there were no positive cases.

Subgroup analysis per site and pathogen group showed a trend toward similar PPAs in Switzerland and Georgia and a tendency toward higher PPA in bacteria compared to virus (Tables S2 and S3). When stratifying based on Ct values with a threshold of 12 in the corresponding LRT samples, AveloMask PPA reached 91% in the low-Ct group and 38% in the high-Ct group (Table S4). A Ct threshold of 12 was selected for exploratory subgroup analyses because for bacterial targets, this threshold corresponded approximately to BioFire-reported bacterial loads of 10⁶ copies/mL in LRT samples, a level that has been associated with a greater likelihood of culture positivity compared with lower loads (19). Because quantitative load measurements are not reported by BioFire for viruses and atypical bacteria, this threshold was applied for all pathogens. Agreement metrics were recalculated using Roche LightMix results from LRT samples as the reference standard, thereby harmonizing the assay methodology. Using this approach, the overall PPA was 63% (95% CI, 51%–73%) and NPA was 100% (95% CI, 99%–100%), with performance estimates similar to those observed when using the BioFire reference standard (Table S5). An analysis of immunocompromised participants (*n* = 11) demonstrated a pathogen distribution comparable to that in the overall cohort. PPA was higher in this subgroup (73%) than in the overall study population (61%); however, the small number of immunocompromised participants does not allow for robust assessment of subgroup-specific performance (Table S6). Among the 13 participants without an acceptable LRT sample, AveloMask samples were available for six participants and detected pathogens in three, including influenza A (*n* = 1) and *S. pneumoniae* (*n* = 3). These findings demonstrate the feasibility of pathogen detection using XBAs in patients unable to provide traditional LRT specimens.

### Comparison with standard-of-care testing

SOCT data were available for 23 participants enrolled in Basel, in whom a total of 81 diagnostic tests were performed (mean, 3.5 tests/participant). Tests included nasopharyngeal swab PCR (*n* = 22), sputum culture (*n* = 15), sputum PCR (*n* = 1), BAL culture (*n* = 3), BAL PCR (*n* = 3), urine antigen test (*n* = 18), blood culture (*n* = 16), and one unspecified test performed on pleural fluid (*n* = 1).

**Fig 4:**
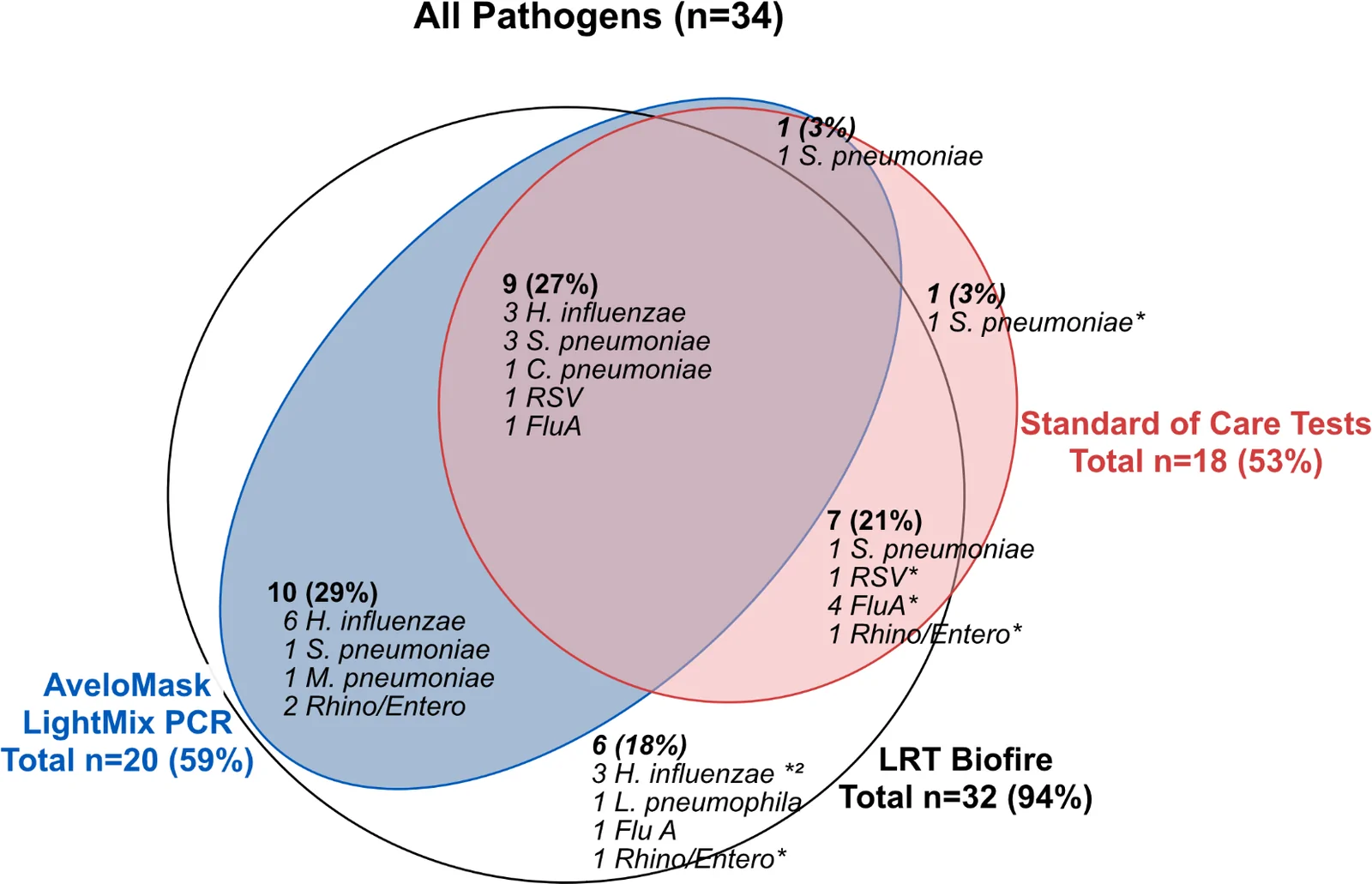
Comparison of pathogen detection by AveloMask LightMix PCR, LRT BioFire, and standard-of-care testing in 23 participants from the Basel study site. Standard-of-care testing included 81 tests (mean, 3.5 tests/participant) including nasopharyngeal swab PCR (*n* = 22), urine antigen test (*n* = 18), blood culture (*n* = 16), sputum culture (*n* = 15), BAL culture (*n* = 3), BAL PCR (*n* = 3), throat/pharyngeal swab PCR (*n* = 2), sputum PCR (*n* = 1), and an unspecified pleural fluid test (*n* = 1). *Co-infecting pathogen not detected by AveloMask LightMix PCR in the presence of a more abundant pathogen. Abbreviation: LRT, lower respiratory tract.

Across AveloMask LightMix, LRT BioFire, and SOCT, 34 pathogens were detected. Detection overlap is illustrated in Fig. 4. AveloMask detected 20/34 pathogens (59%), SOCT 18/34 (53%), and LRT BioFire 32/34 (94%). Relative to SOCT, AveloMask missed eight pathogens, including seven co-infections (six viral [four influenza A, one RSV, and one rhinovirus] and one *S. pneumoniae*) that occurred alongside a more abundant bacterial infection. LRT BioFire detected six additional pathogens that were not detected by AveloMask LightMix PCR or SOCT. AveloMask yield was higher for bacterial than viral pathogens (73% vs 33%) (Fig. S2).

### Potential impact on treatment

In an exploratory review by the principal investigator and one independent medical reviewer, adding AveloMask results, available within 24 h, to SOCT data was judged to have the potential to modify antimicrobial management in up to 43% (10/23) of patients. Both reviewers agreed that antimicrobials could have been modified in seven patients. In three additional patients, one reviewer considered antimicrobial de-escalation appropriate, whereas the other recommended no change. Changes included up to seven de-escalations (e.g., switch to penicillin/amoxicillin following *S. pneumoniae* detection), one treatment discontinuation (lack of bacterial detection and low procalcitonin levels), and one optimization (switch from beta-lactam to targeted atypical coverage following *M. pneumoniae* detection). In one case, the reviewers differed regarding the intervention (discontinuation vs de-escalation), although both agreed that modification was appropriate. In several patients, bacterial targets detected in LRT BioFire samples were not aligned with the antibiotics prescribed, and their clinical relevance remains uncertain.

### User feedback

A total of 83 participants attempted AveloMask sampling during the study, including individuals whose samples were collected prior to insufficient LRT sampling or were later excluded from PCR analysis. Usability was assessed in all participants who underwent mask sampling. Most participants reported a positive experience: 64/83 (77%) rated the procedure as very or somewhat easy, 11 (13%) as neutral (“okay”), and 8 (10%) as somewhat or very difficult. Overall, AveloMask sampling was well tolerated in this population (Fig. S3). The most common challenge associated with AveloMask use was the perception that the wearing time was too long (*n* = 26) (Fig. S4).

## DISCUSSION

In this study of individuals with LRTIs, AveloMask XBA testing with commercial multiplex qPCR consistently identified the dominant pathogen (the pathogen with the lowest Ct value in the comparator LRT specimen) in 85% of participants. This observed dominant-pathogen detection result met the ≥85% performance target described in target product profiles for minimally invasive LRTI diagnostics (20). Notably, AveloMask predominantly missed lower-abundance, coinfecting pathogens, typically occurring alongside a more abundant bacterial infection, supporting that exhaled pathogen abundance may reflect respiratory shedding intensity (12, 21). From a clinical perspective, identifying the predominant pathogen may be sufficient to guide targeted antimicrobial therapy in many pneumonia cases (22).

Breath-based sampling offers several potential advantages over conventional approaches. Sputum collection can be challenging, as we confirmed in this study. Of the 93 participants enrolled, 13 (14%) were excluded due to sputum production issues, in line with other studies showing difficulty in obtaining adequate sputum samples in this population (23–25). Contrastingly, mask sampling was feasible in routine ward settings and well tolerated by hospitalized pneumonia patients, with most participants rating the procedure as easy. These findings support the potential of XBA sampling as a complementary diagnostic approach to increase diagnostic yield, particularly when sputum is unavailable or of poor quality. The present study was not designed to evaluate diagnostic performance in patients unable to provide a suitable sputum specimen; nevertheless, AveloMask detected pathogens in three of six available samples from participants without an acceptable LRT comparator sample, suggesting additional diagnostic yield in this population. Future studies should assess specimen availability and diagnostic yield alongside diagnostic accuracy to better define the role of XBAs in pneumonia diagnostics (26).

The differences between AveloMask and LRT sample results may reflect several factors, including differences in pathogen burden, sampling site, and the potential distinction between colonization and infection, particularly for bacterial pathogens detected using the BioFire Pneumonia Panel (27). Organisms such as *H. influenzae* are frequently detected in LRT samples but may not always represent the primary cause of disease (28). In our study, AveloMask preferentially detected lower-Ct pathogens, suggesting an influence of pathogen abundance. This concept is consistent with prior work highlighting challenges in distinguishing colonization from infection in molecular diagnostics (29, 30). Yet, this study was not designed to determine whether AveloMask detections more closely reflect clinically significant infection than LRT testing; future studies will require dedicated clinical adjudication.

Across targets, the AveloMask Kit achieved an overall PPA of 61%. While lower than the concordance observed for dominant-pathogen detection, this reflects the preferential detection of high-abundance pathogens and reduced detection of lower-abundance, coinfecting organisms. This pattern suggests that discordance is likely driven by biological factors, although technical failure of the AveloMask or the assay cannot be fully excluded. Concordance was higher for bacterial than viral pathogens, and discordance was particularly pronounced for influenza A. The lower PPA for viral pathogens may reflect that many patients with LRTIs present after the peak of viral replication and shedding, when viral abundance is declining despite symptoms. Viral nucleic acid may remain detectable in LRT specimens while XBA concentrations fall below detection thresholds. This is in line with prior studies showing that exhaled viral abundance decreases rapidly over time (31, 32). Differences in collection timing and antimicrobial initiation may also have contributed to some discordant results (33, 34).

The NPA of AveloMask LightMix PCR was 99.8% against LRT BioFire results, with one instance of an AveloMask-positive/LRT-negative result for *S. pneumoniae*. SOCT for this participant, including a positive pneumococcal urine antigen test and sputum culture, supported the presence of pneumococcal infection. This case illustrates that breath sampling can detect pathogens missed in comparator testing and underscores the imperfect nature of any single reference standard. While this suggests high specificity of AveloMask sampling, interpretation is limited by the absence of a universally accepted gold standard for etiological LRTI diagnostics and the potential for molecular testing of LRT samples to detect organisms of uncertain clinical relevance (35).

In participants with available SOCT data, comparison of AveloMask results showed substantial overlap with those from routine diagnostic testing. Conversely, additional LRT BioFire detections sometimes represented coinfections or organisms with unclear clinical significance based on available microbiologic and clinical data. These assessments were exploratory and included instances where organisms detected during LRT BioFire testing were not supported by standard culture findings, were not specifically targeted by antimicrobials, or were identified alongside other pathogens at higher abundance. The assessment suggested that AveloMask results available within 24 h could have influenced antimicrobials in up to 43% of cases, predominantly through de-escalation. Although preliminary, this suggests that XBA pathogen detection may support more targeted antimicrobial use and complement existing diagnostic workflows.

This study has several strengths. First, the paired-sample design enabled direct within-participant comparison between XBA and LRT specimens, reducing confounding from interindividual variability. Second, participants were enrolled using standardized clinical criteria, and comparator samples were restricted to good-quality LRT specimens, ensuring a clinically well-defined cohort. Third, exclusions were systematically reported according to the Standards for Reporting Diagnostic Accuracy Studies (STARD, Table S7). Fourth, the study assessed both performance and user feedback, providing insights into real-world feasibility of XBA sampling in hospitalized patients with LRTIs. Fifth, the BioFire Panel was selected as the primary reference because it is a validated and widely implemented platform for LRT specimens. In addition, residual LRT samples were tested with the same LightMix assay used for AveloMask samples, yielding similar concordance estimates and largely ruling out assay-related reference standard differences.

This study also has limitations. First, the study population may be subject to selection bias because inclusion required availability of a good-quality LRT comparator, which was predominantly sputum. This may limit generalizability to patients unable to expectorate, in whom the clinical utility of XBA sampling may be highest. Second, although enrollment allowed for antibiotic initiation 48 h before XBA sampling, this interval may have reduced XBA bacterial abundance, potentially lowering concordance between breath and LRT samples, particularly as sputum was generally collected before XBA sampling (36–38). Third, low prevalence for several individual pathogens reduced the precision of pathogen-specific estimates. Fourth, a comparison to SOCT data was only possible in participants from Basel, and assessment of performance against culture or a composite reference standard requires further studies. Fifth, SARS-CoV-2 was not included in the LRT BioFire panel and was, therefore, assessed only in the secondary LightMix-based analysis, in which no samples were positive. Sixth, only a single XBA sample was collected from each participant, precluding the assessment of intra-participant variability and reproducibility. Seventh, although extraction and process controls were included, environmental controls were not collected and should be considered in future studies. Finally, sputum quality was assessed using a threshold of <25 squamous epithelial cells per low-power field, and stricter sputum-quality criteria could influence concordance estimates between breath and LRT samples.

### Conclusion

AveloMask breath sampling demonstrated biological feasibility for molecular detection of LRT pathogens and identified the dominant pathogen in matched LRT samples with high concordance. Overall PPA was moderate, largely reflecting reduced detection of low-abundance and coinfecting pathogens, while NPA was 99.8%. The noninvasive procedure was well tolerated in hospitalized pneumonia patients, supporting breath-based sampling as a noninvasive adjunct to conventional LRT diagnostics. Larger studies and careful interpretation of XBA results in the clinical context are warranted to further define its clinical role.

## Data Availability

Data generated or analyzed during this study are included in the article, in the supplemental material, and supplemental data spreadsheet.

## SUPPLEMENTAL MATERIAL

Data S1 jcm.00562-26-s0001.xlsx Participant data.

Supplemental Material jcm.00562-26-s0002.docx Fig. S1 to S4; Tables S1 to S7.

Movie S1 jcm.00562-26-s0003.mp4 Steps to collect and process an AveloMask sample.

ASM does not own the copyrights to Supplemental Material that may be linked to, or accessed through, an article. The authors have granted ASM a non-exclusive, world-wide license to publish the Supplemental Material files. Please contact the corresponding author directly for reuse.
