# Supplementary material for: Breath aerosol PCR for detection of lower respiratory tract infections: evaluation of a non-invasive face mask collector in pneumonia patients
Source: J Clin Microbiol. 2026 Sep 10;64(10):e00562-26. doi: 10.1128/jcm.00562-26 (PMC13712747; doi:10.1128/jcm.00562-26)
Supplement: Supplementary file 1 [file jcm.00562-26-s0002.docx]

**Supplemental Material for:**

**Breath aerosol PCR for detection of lower respiratory tract infections: Evaluation of a non-invasive face mask collector in pneumonia patients**

**Table of Contents**

Movie S1: AveloMask Kit Instructions	2

Figure S1: AveloMask Kit User Instructions	3

Figure S2: Bacteria vs. Virus Detection Overlap in 23 participants from Basel	4

Figure S3: Participant Feedback	5

Figure S4: Participant Challenges	6

Table S1. Reasons for no index sample.	7

Table S2: Per-pathogen and overall concordance between detections for paired LRT samples (Biofire panel) and AveloMask samples (LightMix panel) stratified by study site.	8

Table S3: Per-pathogen and overall concordance between detections for paired LRT samples (Biofire panel) and AveloMask samples (LightMix panel) stratified by bacteria and virus.	9

Table S4: Per-pathogen and overall PPA for paired LRT samples (Biofire panel) and AveloMask samples (LightMix panel) stratified by LRT sample Ct value	10

Table S5: Per-pathogen and overall concordance between detections for paired LRT samples (Lightmix panel) and AveloMask samples (LightMix panel).	11

Table S6: Per-pathogen and overall concordance for paired LRT samples (Biofire panel) and AveloMask samples (LightMix panel) for immunocompromised participants.	12

Table S7: STARD Checklist	13

Data S1: Supplemental Data	15

**Supplemental Figures, Videos, Tables, and Data**

# Movie S1: AveloMask Kit Instructions

The movie shows the steps to collect and process an AveloMask sample.

# Figure S1: AveloMask Kit User Instructions

# Figure S2: Bacteria vs. Virus Detection Overlap in 23 participants from Basel

# Figure S3: Participant Feedback

| **Participant Feedback** | **N=83** |
| --- | --- |
| Ease-of-Use |  |
| Very Easy | 42 (51%) |
| Somewhat Easy | 22 (26%) |
| Okay | 11 (13%) |
| Somewhat Difficult | 4 (5%) |
| Very Difficult | 4 (5%) |

# Figure S4: Participant Challenges

*Study participants could provide more than one perceived challenge. Therefore, percentages add up to more than 100%.

| **Participant Challenges** | **N=83*** |
| --- | --- |
| No aspects are challenging | 34 (41%) |
| Mask wearing time too long | 26 (31%) |
| Mask is uncomfortable | 17 (20%) |
| Too many forced coughs | 14 (17%) |
| Difficulty breathing in mask | 7 (8%) |
| Difficulty coughing in mask | 5 (6%) |
| Unsure | 4 (5%) |
| All aspects are challenging | 1 (1%) |

Table S1. Reasons for no index sample.

# Table S2: Per-pathogen and overall concordance between detections for paired LRT samples (Biofire panel) and AveloMask samples (LightMix panel) stratified by study site.

Abbreviations: LRT, lower respiratory tract; OPA, overall percent agreement; PPA, positive percent agreement; NPA, negative percent agreement; PPV, positive predictive value; NPV, negative predictive value

# Table S3: Per-pathogen and overall concordance between detections for paired LRT samples (Biofire panel) and AveloMask samples (LightMix panel) stratified by bacteria and virus.

Abbreviations: LRT, lower respiratory tract; OPA, overall percent agreement; PPA, positive percent agreement; NPA, negative percent agreement; PPV, positive predictive value; NPV, negative predictive value

# Table S4: Per-pathogen and overall PPA for paired LRT samples (Biofire panel) and AveloMask samples (LightMix panel) stratified by LRT sample Ct value

Abbreviations: LRT, lower respiratory tract; PPA, positive percent agreement; Ct, cycle threshold

# Table S5: Per-pathogen and overall concordance between detections for paired LRT samples (Lightmix panel) and AveloMask samples (LightMix panel).

Abbreviations: LRT, lower respiratory tract; OPA, overall percent agreement; PPA, positive percent agreement; NPA, negative percent agreement; PPV, positive predictive value; NPV, negative predictive value

# Table S6: Per-pathogen and overall concordance for paired LRT samples (Biofire panel) and AveloMask samples (LightMix panel) for immunocompromised participants.

Abbreviations: LRT, lower respiratory tract; OPA, overall percent agreement; PPA, positive percent agreement; NPA, negative percent agreement; PPV, positive predictive value; NPV, negative predictive value

# Table S7: STARD Checklist

| **Section & Topic** | **No** | **Item** | **Done?** |
| --- | --- | --- | --- |
|  |  |  |  |
| **TITLE OR ABSTRACT** |  |  |  |
|  | **1** | Identification as a study of diagnostic accuracy using at least one measure of accuracy  (such as sensitivity, specificity, predictive values, or AUC) | yes |
| **ABSTRACT** |  |  |  |
|  | **2** | Structured summary of study design, methods, results, and conclusions  (for specific guidance, see STARD for Abstracts) | yes |
| **INTRODUCTION** |  |  |  |
|  | **3** | Scientific and clinical background, including the intended use and clinical role of the index test | yes |
|  | **4** | Study objectives and hypotheses | yes |
| **METHODS** |  |  |  |
| *Study design* | **5** | Whether data collection was planned before the index test and reference standard  were performed (prospective study) or after (retrospective study) | yes |
| *Participants* | **6** | Eligibility criteria | yes |
|  | **7** | On what basis potentially eligible participants were identified  (such as symptoms, results from previous tests, inclusion in registry) | yes |
|  | **8** | Where and when potentially eligible participants were identified (setting, location and dates) | yes |
|  | **9** | Whether participants formed a consecutive, random or convenience series | yes |
| *Test methods* | **10a** | Index test, in sufficient detail to allow replication | yes |
|  | **10b** | Reference standard, in sufficient detail to allow replication | yes |
|  | **11** | Rationale for choosing the reference standard (if alternatives exist) | yes |
|  | **12a** | Definition of and rationale for test positivity cut-offs or result categories  of the index test, distinguishing pre-specified from exploratory | yes |
|  | **12b** | Definition of and rationale for test positivity cut-offs or result categories  of the reference standard, distinguishing pre-specified from exploratory | yes |
|  | **13a** | Whether clinical information and reference standard results were available  to the performers/readers of the index test | yes |
|  | **13b** | Whether clinical information and index test results were available  to the assessors of the reference standard | yes |
| *Analysis* | **14** | Methods for estimating or comparing measures of diagnostic accuracy | yes |
|  | **15** | How indeterminate index test or reference standard results were handled | yes |
|  | **16** | How missing data on the index test and reference standard were handled | yes |
|  | **17** | Any analyses of variability in diagnostic accuracy, distinguishing pre-specified from exploratory | yes |
|  | **18** | Intended sample size and how it was determined | yes |
| **RESULTS** |  |  |  |
| *Participants* | **19** | Flow of participants, using a diagram | yes |
|  | **20** | Baseline demographic and clinical characteristics of participants | yes |
|  | **21a** | Distribution of severity of disease in those with the target condition | yes |
|  | **21b** | Distribution of alternative diagnoses in those without the target condition | no |
|  | **22** | Time interval and any clinical interventions between index test and reference standard | yes |
| *Test results* | **23** | Cross tabulation of the index test results (or their distribution)  by the results of the reference standard | yes |
|  | **24** | Estimates of diagnostic accuracy and their precision (such as 95% confidence intervals) | yes |
|  | **25** | Any adverse events from performing the index test or the reference standard | yes |
| **DISCUSSION** |  |  |  |
|  | **26** | Study limitations, including sources of potential bias, statistical uncertainty, and generalisability | yes |
|  | **27** | Implications for practice, including the intended use and clinical role of the index test | yes |
| **OTHER INFORMATION** |  |  |  |
|  | **28** | Registration number and name of registry | yes |
|  | **29** | Where the full study protocol can be accessed | no |
|  | **30** | Sources of funding and other support; role of funders | yes |
|  |  |  |  |

# Data S1: Supplemental Data

Participant data
